# Mechanism of impaired microtubule-dependent peroxisome trafficking and oxidative stress in *SPAST*-mutated cells from patients with Hereditary Spastic Paraplegia

**DOI:** 10.1038/srep27004

**Published:** 2016-05-27

**Authors:** Gautam Wali, Ratneswary Sutharsan, Yongjun Fan, Romal Stewart, Johana Tello Velasquez, Carolyn M Sue, Denis I. Crane, Alan Mackay-Sim

**Affiliations:** 1 Eskitis Institute for Drug Discovery, Griffith University, Brisbane, Australia; 2 Kolling Institute of Medical Research, University of Sydney, Sydney, Australia; 3 The University of Queensland Centre for Clinical Research, Brisbane, Australia

## Abstract

Hereditary spastic paraplegia (HSP) is an inherited neurological condition that leads to progressive spasticity and gait abnormalities. Adult-onset HSP is most commonly caused by mutations in *SPAST*, which encodes spastin a microtubule severing protein. In olfactory stem cell lines derived from patients carrying different *SPAST* mutations, we investigated microtubule-dependent peroxisome movement with time-lapse imaging and automated image analysis. The average speed of peroxisomes in patient-cells was slower, with fewer fast moving peroxisomes than in cells from healthy controls. This was not because of impairment of peroxisome-microtubule interactions because the time-dependent saltatory dynamics of movement of individual peroxisomes was unaffected in patient-cells. Our observations indicate that average peroxisome speeds are less in patient-cells because of the lower probability of individual peroxisome interactions with the reduced numbers of stable microtubules: peroxisome speeds in patient cells are restored by epothilone D, a tubulin-binding drug that increases the number of stable microtubules to control levels. Patient-cells were under increased oxidative stress and were more sensitive than control-cells to hydrogen peroxide, which is primarily metabolised by peroxisomal catalase. Epothilone D also ameliorated patient-cell sensitivity to hydrogen-peroxide. Our findings suggest a mechanism for neurodegeneration whereby *SPAST* mutations indirectly lead to impaired peroxisome transport and oxidative stress.

Mutations in *SPAST* are the most common cause of autosomal-dominant, adult-onset hereditary spastic paraplegia (HSP), which is defined clinically by lower limb spasticity and paralysis characterised by degeneration of the corticospinal tract^1^,^2^. Widespread involvement of the corticospinal white matter tracts are also seen in subclinical patients with *SPAST* mutations as measured by MRI and diffusion tensor imaging^3^,^4^. White matter losses can be observed at the whole brain level and in frontal and temporal lobes, cerebellum, and other regions in some HSP patients with and without *SPAST* mutations^3^,^4^,^5^,^6^. These observations suggest that axonal loss may be more widespread throughout the central nervous system in HSP and not just confined to the long axons of the corticospinal tract upon which diagnosis is dependent. The consequences of *SPAST* mutations may be evident in most cells but amplified in neurons with long axons.

*SPAST* encodes spastin, which severs stabilised microtubules that are required for intracellular organelle transport^7^. Mouse neurons carrying mutations in *SPAST/SPG4* had reduced anterograde transport of mitochondria^8^,^9^,^10^ and human neurons carrying *SPAST* mutations had reduced retrograde transport of mitochondria^11^,^12^. Human olfactory neural stem cells with *SPAST* mutations have impaired transport of peroxisomes^13^. Peroxisomes are essential organelles that are involved in the responding to oxidative stress, particularly in metabolism of hydrogen peroxide^14^. In patient cells with heterozygous *SPAST* mutations there were reduced levels of acetylated α-tubulin, a marker for stabilised microtubules, and reduced speeds of peroxisome transport both of which were restored to control levels by low doses of several tubulin-binding drugs^15^.

One aim of the present study is to understand the cellular mechanism that reduced the average speed of peroxisome transport in patient-derived cells compared to control-derived cells. Two hypothetical mechanisms suggest themselves. The first is that movement of individual peroxisomes is slowed by impairment of the interaction between individual peroxisomes and the stabilised microtubules, which would slow down individual peroxisomes thereby reducing the average speed of the population. The peroxisome-microtubule interaction was observed indirectly from the time-dependent dynamics of movement of individual peroxisomes. Not all peroxisome movement is microtubule-dependent. Two strategies ensured that only microtubule-dependent movement was assessed: first, analysis concentrated on the fastest moving group of peroxisomes; and second, experiments were confined to cell processes with microtubules but no actin cytoskeleton that could interfere with microtubule dynamics and interactions, as pertains in axons. The second mechanism that could reduce the average speed of peroxisome movement in patient cells would be a reduction in the availability of stabilised microtubules upon which peroxisomes can travel. Patient cells have less acetylated α-tubulin than control cells, indicating fewer stabilised microtubules. This could reduce the probability of peroxisome-microtubule interactions and restrict the number of peroxisomes being able to move along microtubules thereby reducing the average speed of the peroxisome population. This mechanism was assessed by comparing the numbers of peroxisomes moving at different speeds, with an emphasis on the fastest group of peroxisomes, those whose movement is unequivocally microtubule-dependent.

In many neurodegenerative diseases the proximate cause of neuronal death is thought to be oxidative stress but this has not been investigated in *SPAST*-mediated HSP. Peroxisomes and mitochondria regulate the redox state of cells and protect against oxidative stress^16^ so impairments in microtubule-dependent transport could potentially induce oxidative stress in *SPAST*-mutated cells by altering peroxisome and mitochondria distributions and functions. The second aim of the present study was to investigate hydrogen peroxide-induced oxidative stress in patient-derived cells carrying *SPAST* mutations and to test whether this was dependent on microtubule-dependent organelle transport. The prediction was that impaired transport of peroxisomes would make patient-derived cells more sensitive to hydrogen peroxide and that epothilone D would restore oxidative stress to control levels by restoring peroxisome transport. Peroxisomes may play the critical role here because detoxification of hydrogen peroxide is predominantly performed by peroxisomal catalase, with a much lesser contribution from mitochondrial glutathione peroxidase and other enzymes^17^.

## Results

### Axon-like processes were generated by differentiation of ONS cells

**Figure 1:**
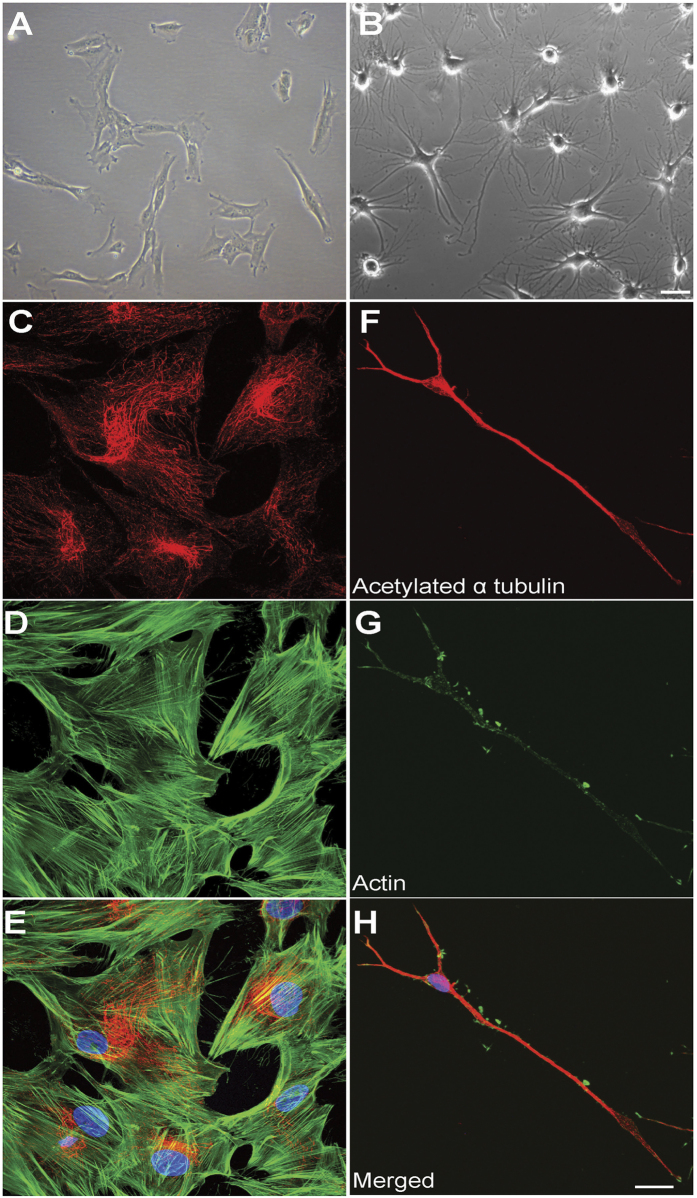
Axon-like processes induced by neuronal differentiation of ONS cells. (**A**) Phase-contrast image of control ONS cells in normal growth medium. (**B**) Phase contrast image of control ONS cells grown in cytochalasin (**D**) neuron-induction medium to generate immature neurons with axon-like processes. (**C–E**) The same field of undifferentiated ONS cells labelled with an antibody to acetylated α-tubulin (red, **C**) and phalloidin labelling of actin (green, **D**), and merged with nuclei labelled with DAPI (blue, **E**). (**F–H**) The same field showing a differentiated cell labelled with an antibody to acetylated α-tubulin (red, **F**) and phalloidin labelling of actin (green, **G**), and merged with nuclei labelled with DAPI (blue, **H**). In ONS cells microtubules and actin filaments overlap. Differentiated cells have microtubules but the actin filaments are depleted. Scale bar in (**B**) applies to (**A,B**) 100 μm. Scale bar in (**H**) applies to (**C–H**) 20 μm.

Olfactory neurosphere-derived stem cells (ONS cells) were derived from nasal biopsies of patients and healthy controls as described previously^13^,^18^. Undifferentiated ONS cells are flat with multiple short processes (Fig. 1A) and complex networks of microtubules (acetylated α-tubulin labelled; Fig. 1C) and actin filaments (phalloidin labelled; Fig. 1D) distributed throughout the cytoplasm (Fig. 1E). After neuronal induction and treatment with cytochalasin D, ONS cells differentiated into multipolar and bipolar cells containing elongated, thin neurites with lengths of 150–300 μm and diameters of 0.5–1 μm (Fig. 1B). Microtubules in these differentiated cells extended the length of the long-thin processes resembling the microtubule arrays of axons (Fig. 1F). In contrast, the actin filament network in differentiated cells was severely disrupted; with actin visible as clumps in the cytoplasm (Fig. 1G) and no association with the microtubule network (Fig. 1H). This cytoskeletal arrangement allowed us to investigate microtubule-dependent organelle transport without the influence of actin. For this reason the neurites are referred to as “axon-like processes”. The control- and patient-derived cells were affected similarly by the differentiation procedure.

### Peroxisome distribution in axon-like processes

**Figure 2:**
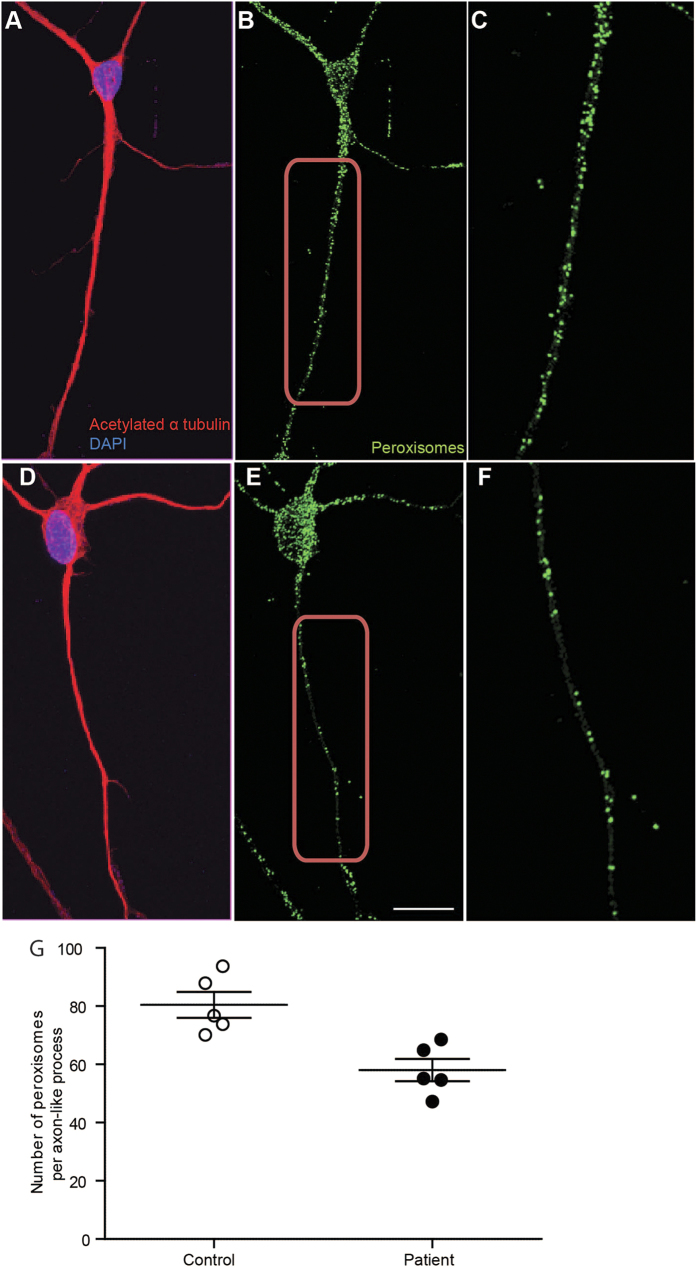
Reduced peroxisome number in axon-like processes of differentiated patient cells. (**A**) Control differentiated cells immunostained with an antibody to acetylated α-tubulin (red), and DAPI to label nuclei (blue). (**B**) The same cell immunostained with an antibody to the peroxisomal membrane protein PEX14 (green). (**C**) Higher magnification view of the boxed area in (**B**), showing the individual peroxisomes in axon-like process. (**D**) Patient differentiated cell immunostained with an antibody to acetylated α-tubulin (red), and with DAPI (blue). (**E**) The same cell immunostained with an antibody to PEX14 (green). (**F**) Higher magnification view of the boxed area in (**E**), showing the individual peroxisomes in the axon-like process. Scale bar in E applies to (**A,B,D,E**) 20 μm. (**G)** Peroxisome numbers were quantified along 100 μm of axon-processes starting 20 μm from cell body (example shown in **C,F**). 4022 peroxisomes were quantified from 50 axon-like processes of 5 control cell lines and 2903 peroxisomes were quantified from 50 axon-like processes of 5 patient cell lines. Average numbers of peroxisomes/axon-process of all patient cell lines (solid circles) were compared to all control cell lines (open circles). N = 5 cell lines per group; unpaired t-test; two tailed; p-value: 0.0052; t = 3.808 df = 8. Data are represented as Mean ± SEM.

In differentiated control cells, peroxisomes were distributed throughout the cell body and along the axon-like processes (Fig. 2A–C). In differentiated patient cells, peroxisomes were fewer along the axon-like processes (Fig. 2D–F). Patient cells had 25% fewer peroxisomes along the axon-like processes compared to control cells (Fig. 2G; peroxisomes/control axon-like processes: 80.4 ± 4.46, peroxisomes/patient cell processes: 58.06 ± 3.83, t = 3.808; df = 8; p < 0.01).

### Time-dependent dynamics of peroxisome movement in axon-like processes cells

**Figure 3:**
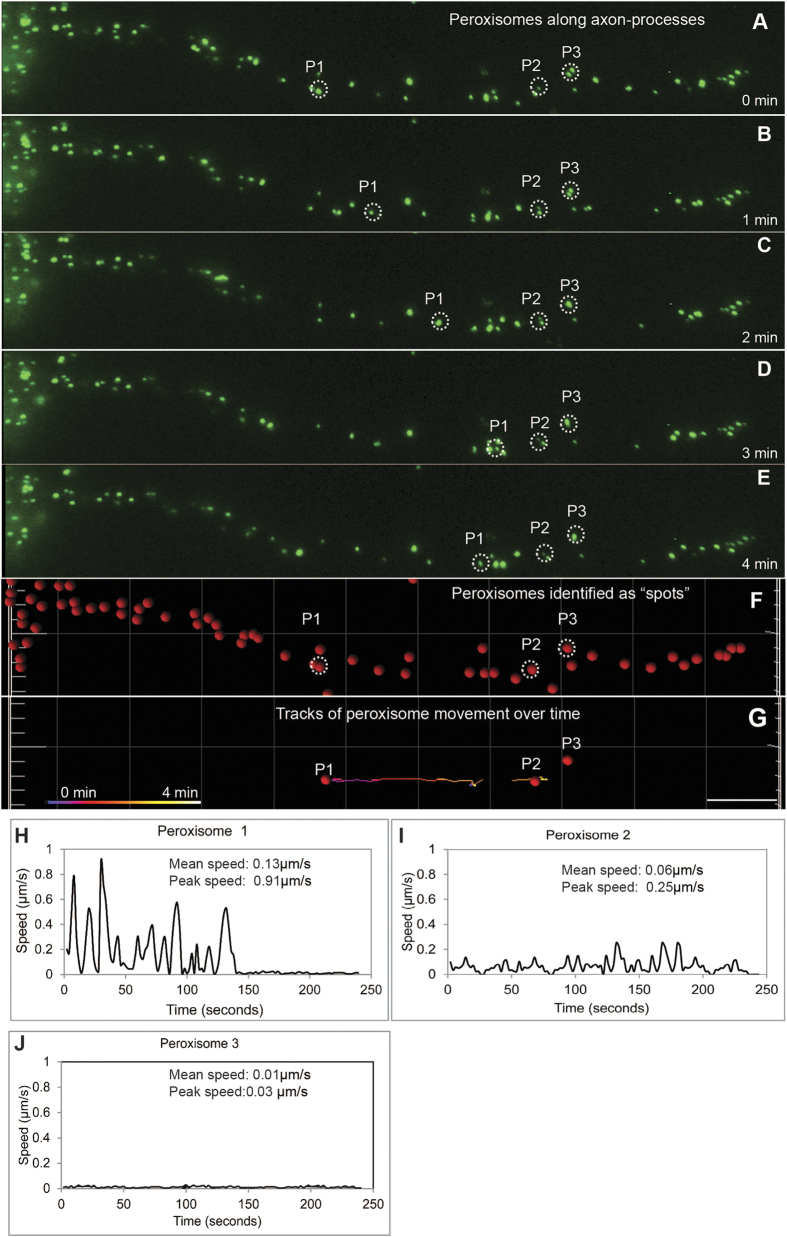
Automated analysis of peroxisome movement along axon-like processes. (**A–E**) Images from a time-lapse movie of a control axon-like process at the times indicated. GFP-labelled peroxisomes are evident (green dots). Three example peroxisomes (P1-P3, circled) indicate peroxisomes moving different distances during the four minutes. (**F**) The computer-generated representation of the peroxisomes in the image shown in A (red dots). (**G**) The computer-generated tracks of movement of P1-P3 assessed from successive 2 second images over the 4 minute observation period. Scale bar in (**G**) applies to (**A–G**) 10 μm. (**H–J**) The speed of peroxisome movement was plotted against time for the three identified peroxisomes. At this time scale, peroxisome movement was characterised by saltatory movement with bursts of movement separated by rest periods. The fastest moving peroxisomes were characterised by multiple fast saltatory movements with rest periods, as for peroxisome P1. The majority of peroxisomes moved either like peroxisome P2, with multiple slow saltatory movements characterised as Brownian-like motion, or were immobile like peroxisome P3.

Peroxisome movement along the axon-like processes was quantified using time-lapse imaging of GFP-labelled peroxisomes. Figure 3A–E is a series of images at 1 minute intervals showing the peroxisomes (green spots) in a control axon-like process. Three example peroxisomes (P1, P2 and P3) are circled showing their positions over the four minute observation period (Fig. 3A–E). Figure 3F is the output of the image analysis corresponding to the image in Fig. 3A, showing all identified peroxisomes (red spots) and the three example peroxisomes circled. Figure 3G shows the paths of movement of the three identified peroxisomes during the observation period. The image analysis software computed paths for all identified peroxisomes. For a detailed view of time-dependent movement dynamics, peroxisome speeds were plotted against time (Fig. 3H–J). The three example peroxisomes illustrate different dynamics during the observation period (Fig. 3H–J). P1 showed many bursts of fast saltatory movement followed by inactivity. P2 had frequent bursts of slower saltatory movement, while P3 did not move at all during the observation period. Average speeds of peroxisome movement and distance travelled were calculated for the whole observation period. Most peroxisomes (~85–90%) travelled slowly (e.g. P2 and P3), achieving only short distances. A minority of peroxisomes (10–15%) travelled at fast speeds, achieving longer distances during the observation period (e.g. P1). These characteristics of peroxisome movement are universal to most mammalian cells, with the fast transport of peroxisomes being microtubule-dependent^19^. Both fast and slow moving peroxisomes were observed in patient and control cells.

### Saltatory movement of peroxisomes in axon-like processes cells

**Figure 4:**
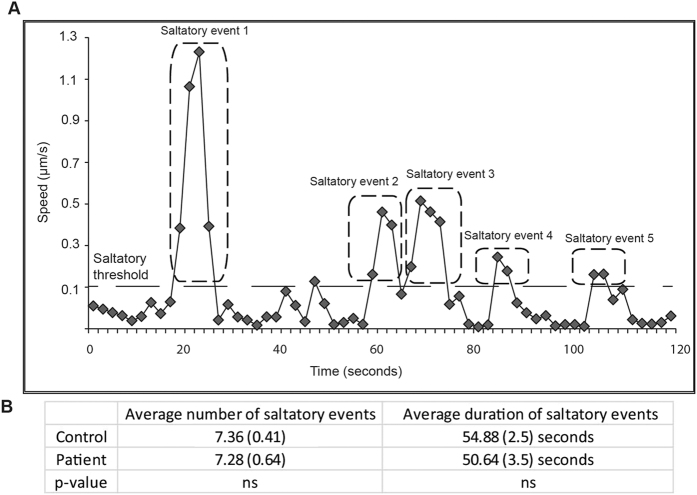
Time-dependent dynamics of peroxisome movement. (**A**) Example of saltatory movements of a single peroxisome from a patient axon-like process, with speed plotted against time. Saltatory events were defined as those exceeding a threshold (dashed line at 0.1 μm/s). Five saltatory events are indicated. (**B**) Saltatory events were quantified from approximately 200 fast moving peroxisomes from patient and control axon-like processes. Data are represented as Mean ± SEM. Fast moving peroxisomes were defined as those exceeding the average speed of the fastest 10% of control peroxisomes (1.4 μm/s).

Saltatory movements were analysed in the fast moving peroxisomes in axon-like processes of patient and control cells. “Fast moving” peroxisomes were defined as those with average speed greater than 0.14 μm/s, the speed of the fastest 10% of control peroxisomes. A saltatory event was one exceeding 0.1 μm/s (threshold shown as dashed line, Fig. 4A). Fast moving peroxisomes displayed multiple saltatory events of varied durations and speeds (Fig. 4A). There was no difference between saltatory movements of fast moving peroxisomes in control-derived and patient-derived axon-like processes, either in the average number of saltatory events or their duration (Fig. 4B).

### The number of fast moving peroxisomes was reduced in axon-like processes of patient cells

**Figure 5:**
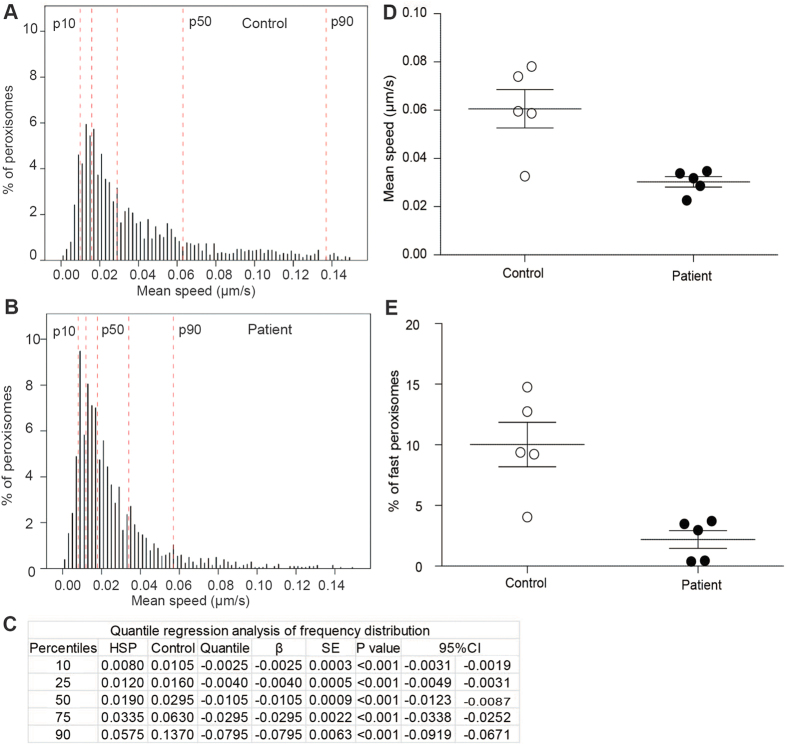
Reduced numbers of fast moving peroxisomes in patient cell axon-like processes. (**A,B**) Frequency distributions of peroxisome speeds in populations of peroxisomes from control (**A**; N = 2837 peroxisomes from 50 axon-like processes of 5 control cell lines) and patient axon-like processes (**B**; N = 2023 peroxisomes from 50 axon-like processes of 5 patient cell lines). The red dotted lines indicate the 10th, 25th, 50th, 75th and 90th percentiles of the frequency distribution. Compared to the control peroxisomes, the frequency distribution of the patient peroxisomes is shifted to the left, indicating a shift to more slowly moving peroxisomes. (**C**) Quantile regression analysis of the frequency distributions, showing a significant reduction at all percentiles in mean peroxisome speeds in patient cells (HSP) compared to control cells. For inter-percentile comparisons the patient-control difference (β), standard error (SE), significance (p-value) and 95% confidence interval (CI) values are shown. (N = 2837 peroxisomes from 50 control axon-like processes of 5 control cell lines and 2023 peroxisomes from 50 control axon-like processes of 5 control cell lines). (**D**) Mean speeds of peroxisomes travelling along patient and control axon-like processes. 2837 peroxisomes from 50 control axon-like processes of 5 control cell lines and 2023 peroxisomes from 50 control axon-like processes of 5 control cell lines were analysed. Average mean speeds of peroxisomes of all patient cell lines (solid circles) were compared to all control cell lines. N = 5 cell lines per group. Unpaired t-test; two tailed; p-value: 0.0064; t = 3.663 df = 8. Data are represented as Mean ± SEM. (**E**) Average percentage of fast moving peroxisomes of all patient cell lines (solid circles) were compared to all control cell lines (open circles). N = 5 cell lines per group. Unpaired t-test; two tailed; p-value: 0.0041; t = 3.975 df = 8. Data are represented as Mean ± SEM.

The speeds of peroxisome movement were calculated for approximately 5000 peroxisomes in axon-like processes of differentiated cells from five controls and five patients. Frequency distributions of the populations of peroxisome speeds demonstrated that the patient peroxisomes moved more slowly than control peroxisomes (Fig. 5A,B). As an illustration of the population differences, the 90th percentile of patient peroxisomes had approximately the same mean speed as the 75th percentile of the control peroxisomes (Fig. 5A,B). In effect, the patient speed frequency distribution was shifted to the left across the whole population. The populations were compared statistically by quantile regression (Fig. 5C). This demonstrated that patient peroxisome speeds were significantly slower than control peroxisomes in each percentile (p < 0.001). This difference in peroxisome speeds was reflected in the speeds of peroxisomes averaged for the five patient-derived and five control-derived cells (Fig. 5D; control: 0.06 ± 0.002 μm/s; patient: 0.03 ± 0.001 μm/s; t = 3.659; df = 8; p < 0.01). The percentage of fast moving peroxisomes in control axon-like processes was significantly more than in patient axon-like processes (Fig. 5E; control: 10% ± 1.83; patient: 2.3% ± 0.73; t = 3.975; df = 8; p < 0.01).

The anterograde or retrograde direction of movement of fast moving peroxisomes was quantified from visual analysis of 50 patient and 50 control axon-like processes. The percentages of peroxisomes moving anterogradely and retrogradely in control and patient cells were significantly different (control: 60.9% ± 4.4 and 39.1% ± 4.3, respectively, n = 41; patient: 86.1% ± 6.1 and 13.9% ± 6.2, respectively, n = 30; Chi-square = 59.70, df = 4, p < 0.01). These observations suggest a shift from retrograde to anterograde transport in the patient cells.

### Patient cells were under oxidative stress and were more sensitive to hydrogen peroxide

**Figure 6:**
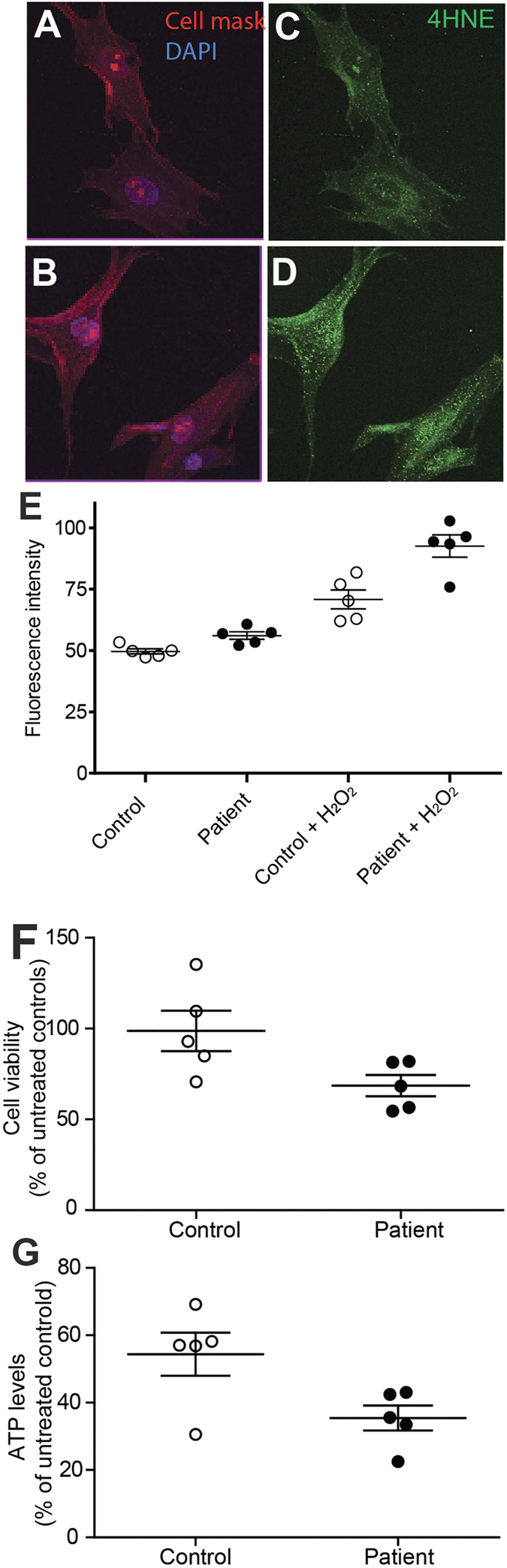
Patient cells were under oxidative stress. (**A**,**B**) Control (**A**) and patient (**B**) ONS cells (cytoplasm, red, Cell mask; nucleus, DAPI, blue). (**C,D**) Control (**C**) and patient (**D**) ONS cells immunostained for the oxidative stress marker (4HNE, green). (**E**) 4HNE fluorescence in cells cultured under baseline conditions (Control, Patient) and 1 hour after 50 μm hydrogen peroxide (Control + H_2_O_2_, Patient + H_2_O_2_). N = 1977 control cells at baseline; 1027 control cells post H_2_O_2_ exposure; 2022 patient cells at baseline and 1003 patient cells post H_2_O_2_ exposure. Average 4HNE fluorescence values of all patient cell lines (solid circles) were compared to all control cell lines (open circles). The fluorescence intensity was significantly different among the groups (repeated measures ANOVA; control v/s patient: p = 0.002; effect of hydrogen peroxide: p < 0.001 and interaction between disease status and treatment: p = 0.042). (**F**) Quantification of cell viability with MTS assay. Average cell viability values measured by MTS assay for all patient cell lines (solid circles) were compared to all control cell lines (open circles). N = 5 cell lines per group. Unpaired t-test; two tailed; p-value: 0.0429; t = 2.404 df = 8. Data are represented as Mean ± SEM. (**G**) Quantification of ATP production with ATPlite assay. Average ATP values measured by ATPlite assay for all patient cell lines (solid circles) were compared to all control cell lines (open circles). N = 5 cell lines per group. Unpaired t-test; two tailed; p-value: 0.0336; t = 2.562 df = 8. Data are represented as Mean ± SEM.

ONS cells were assessed for oxidative stress by immunolabelling for 4-hydoxy-2-nonenal (4HNE) a protein expressed during oxidative stress^20^, under baseline culture conditions and after exposure to H_2_O_2_. Fluorescence of 4HNE was brighter in patient ONS cells compared to control ONS cells grown under standard culture conditions (Fig. 6). Cells were stained with CellMask to define the cell cytoplasm (Fig. 6A,B) and immunostained with an antibody to 4HNE (Fig. 6C,D). Patient cells (Fig. 6D) showed increased 4HNE fluorescence compared to control cells (Fig. 6C). Quantification using automated image analysis, demonstrated significantly higher mean 4HNE fluorescence for the patient cells (Fig. 6E; control: 49.67 ± 1.07; patient: 56.07 ± 1.53). Exposure to hydrogen peroxide increased 4HNE immunofluorescence in control and patient cells, more pronounced for the patient cells (Fig. 6E; control: 70.79 ± 3.85; patient: 92.58 ± 4.50). A repeated measures analysis of variance demonstrated a significant main effect for disease status (control vs patient) (F1,8 = 21.58; p = 0.002) and a significant main effect for hydrogen peroxide treatment (F1,8 = 82.22; p < 0.001). The significant interaction between disease status and treatment (F1,8 = 5.87; p = 0.042) is consistent with the patient cells being significantly more sensitive to hydrogen peroxide treatment than the control cells.

The sensitivity to hydrogen peroxide-induced oxidative stress was further tested using the MTS cell viability assay (Fig. 6F) and the cellular ATP levels (Fig. 6G). Fewer patient-derived cells survived the treatment with hydrogen peroxide demonstrated by the MTS assay (Fig. 6F; control: 98.71 ± 11.09, patient: 68.57 ± 5.85; t = 2.404, df = 8; p < 0.05). In the surviving cells, ATP synthesis was lower in patient cells after hydrogen peroxide treatment (Fig. 6H; control: 54.35 ± 6.38; patient: 35.41 ± 3.79; t = 2.562, df = 8; p < 0.05).

### Epothilone D rescued hydrogen peroxide-induced oxidative stress in patient cells

**Figure 7:**
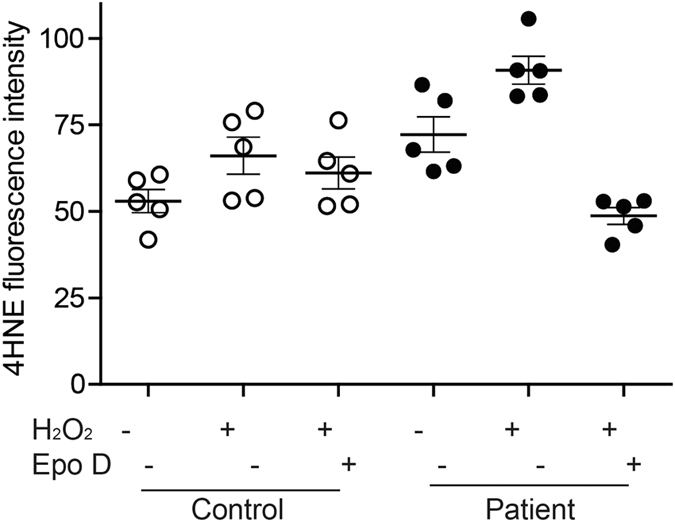
Epothilone D ameliorated hydrogen peroxide-induced oxidative stress in patient cells. 4HNE fluorescence in cells (control and patient) cultured under normal culture conditions; exposed to H_2_O_2_ and treated with 2 nM epothilone D for 7 days and then exposed to H_2_O_2_. N = 1204 control cells under normal culture conditions; 1402 control cells exposed to H_2_O_2_; 1066 control cells treated with epothilone D and exposed to H_2_O_2_; 1178 patient cells under normal culture conditions; 1209 patient cells exposed to H_2_O_2_ and 1013 patient cells treated with epothilone D and exposed to H_2_O_2_. The fluorescence intensity was significantly different among the groups (repeated measures ANOVA; control v/s patient: p = 0.024; main effect of treatment (both H_2_O_2_ and epothilone D): p = 0.008; disease status and treatment: p = 0.007).

Epothilone D (2 nM) restores acetylated α-tubulin levels and the mean speed of peroxisome movement in patient-derived ONS cells^15^. Patient and control ONS cells were grown for 7 days in medium containing 2 nM epothilone D after which they were exposed to hydrogen peroxide (Fig. 7). 4HNE immunofluorescence was quantified at Day 0 (baseline, without treatment with hydrogen peroxide) and at Day 7 after exposure to hydrogen peroxide (with and without treatment with 2 nM epothilone D). As above, patient cells had higher levels of 4HNE immunofluorescence at baseline (control: 52.95 ± 3.35; patient: 72.22 ± 5.09) and after exposure to hydrogen peroxide (control: 66.09 ± 5.41; patient: 90.79 ± 4.05). Cells exposed to epothilone D for 7 days were less sensitive to hydrogen peroxide. After hydrogen peroxide and epothilone D treatment, the 4HNE fluorescence in patient cells was reduced to the baseline level of control cells. In general control cells were less affected by hydrogen peroxide and epothilone D.

A repeated measures analysis of variance showed a significant main effect for disease status (F1,8 = 7.75; p = 0.024) and a significant main effect for treatment, representing both hydrogen peroxide and epothilone D treatments (F2,7 = 10.42; p = 0.008). The significant interaction between disease status and treatment (F2,7 = 11.05; p = 0.007) confirms that the patient cells were significantly different in response to treatments than the control cells.

## Discussion

We show here that microtubule-dependent peroxisome transport is severely impaired, along several dimensions, in axon-like processes in HSP patient cells with *SPAST* mutations. These neurites were “axon-like” in having no actin cytoskeleton so that peroxisome transport was solely dependent on microtubules. The peroxisome transport deficit is much greater under these conditions compared to undifferentiated ONS cells with an intact actin cytoskeleton^13^. In the undifferentiated ONS cells, the mean peroxisome speed in patient cells was lower by about 10% compared to control cells^13^. In the present study, similar analysis performed along microtubules unaffected by actin cytoskeleton in axon-processes of differentiated cells shows that the mean peroxisome speed in patient cells is lower by about 50% compared to control cells. In patient cells axon-like processes there were significantly fewer peroxisomes, of which significantly more were immotile. Additionally there were significantly fewer fast moving peroxisomes; significantly more peroxisomes travelling at slow speeds; and significantly fewer peroxisomes moving retrogradely. In contrast to these differences in *numbers* of transported peroxisomes, there was no patient-control difference in the time-dependent *dynamics* that characterises the saltatory movement of fast microtubule-dependent peroxisomes. These normal dynamics suggest that peroxisome interactions with microtubules and their motor proteins were not affected by *SPAST* mutations. We conclude therefore that *SPAST* mutations affect the *efficiency* of microtubule-dependent peroxisome transport, without affecting the *mechanism* of the saltatory movement. The simplest explanation is that the loss of stabilised microtubules in patient cells reduces the *probability of interaction* between microtubules and peroxisomes. When the number of stabilised microtubules was restored with epothilone D, the numbers of fast moving peroxisomes returned to normal leading to recovery of the average speed of the peroxisome population^15^. Since organelle transport in axons is dependent on the microtubule cytoskeleton^21^, similar mechanisms as those observed here are likely to affect corticospinal axons in patients.

**Figure 8:**
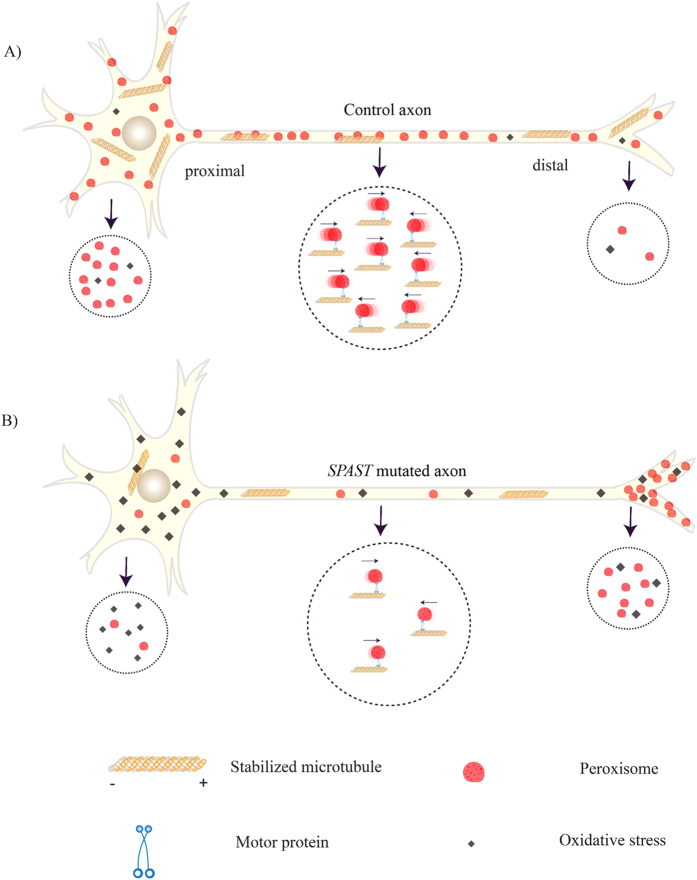
Model for oxidative stress linked to peroxisome trafficking. In healthy axons an adequate microtubule network allows normal numbers of peroxisomes to travel at normal speeds in anterograde and retrograde directions (**A: large circle**). In *SPAST*-mutated axons the reduced microtubule network reduces the number of peroxisomes causing fewer peroxisomes to travel more slowly, with a larger effect on retrograde transport (**B: large circle**). In healthy neurons the peroxisome transport is enough to assure normal peroxisome distributions in cell body and distal axon and appropriate redox state (**A: small circles**). In *SPAST*-mutated neurons impaired peroxisome transport causes a build-up of peroxisomes at the distal end of the axon, with reduced peroxisome turnover at the cell body leading to oxidative stress (**B: small circles**).

Peroxisomes are essential for maintaining axonal integrity^22^. They are transported anterogradely to the distal parts of the axon (up to 1 metre away) and retrogradely back to the cell body for recycling^23^. In *SPAST*-mutated patient cells, there were 25% fewer motile peroxisomes than in control cells and the motile peroxisomes travelled at half the average speed of control peroxisomes. *SPAST*-mutated patient cells accumulate peroxisomes distally^13^. If these cell pathologies occurred in corticospinal motor neurons with 1m long axons, patient peroxisomes would take 9 hr to travel from cell body to distal synapse, compared to 4.5 hr for control peroxisomes. The retrograde journey would be similarly slow but peroxisomes would accumulate distally and fewer peroxisomes would return to the cell body (down from 40% of peroxisomes in control cells to 14% of peroxisomes in patient cells). This set of circumstances would lead to inefficient redox regulation at the distal ends of axons as “old” peroxisomes accumulate compromising their redox buffering capabilities. Normally peroxisomes undergo “pexophagy”, an autophagy-related process, with a half-life of 2 days and a daily fractional turnover rate estimated at approximately 30% 24. Pexophagy involves specialised phagosomes and lysosomal degradation in the cell body close to the nucleus^23^. The deficit in retrograde transport of peroxisomes and their distal accumulation in patient cells would reduce peroxisome turnover and interfere with their ability to regulate pexophagy. In distal axons of *SPAST*-mutated neurons, the average age of peroxisomes would increase and interfere with normal synaptic function by decreasing the local buffering of reactive oxygen species. This could lead to axonopathy and eventually neuronal death (Fig. 8).

Peroxisomes are important regulators of oxidative state in cells; they play central roles in lipid peroxidation and are primarily responsible for hydrogen peroxide metabolism^25^,^26^,^27^. We show here that *SPAST*-mutated patient cells are under oxidative stress, compared to control cells, and more sensitive to hydrogen peroxide. The oxidative stress marker, 4HNE, is produced by lipid peroxidation and is involved in the pathogenesis in several diseases^28^,^29^. In patient cells there was a significant increase in the expression of 4HNE, under baseline culture conditions and after exposure to hydrogen peroxide, which was more toxic to patient cells, assessed by ATP production and cell viability assays. Oxidative stress in patient cells was dependent on stabilised microtubule availability as demonstrated by epothilone D, which restores acetylated α-tubulin levels and peroxisome transport speeds to control levels^15^: epothilone D, restored sensitivity to hydrogen-peroxide oxidative stress in patient cells. Thus, oxidative stress was secondary to the deficit in acetylated α-tubulin level in *SPAST*-mutated HSP patient cells. Our working hypothesis is that the peroxisome transport deficit causes compromised peroxisome function leading to oxidative stress. A central role for peroxisomes is not unprecedented considering that other neurodevelopmental and neurodegenerative disorders (Zellweger syndrome spectrum disorders) arise from disorders of peroxisome biogenesis^30^.

Deficits in microtubule-associated proteins and impaired organelle transport are emerging as common mechanisms in several motor neuron diseases^31^ while chronic oxidative stress is seen as another mechanism for neuronal loss in neurodegenerative diseases^14^,^26^,^30^. Our evidence in *SPAST*-mutated cells demonstrates a direct link between these mechanisms showing that both are restored with epothilone D, which normalises the microtubule cytoskeleton. This mechanism may also apply to mitochondria whose transport is impaired in patient-derived *SPAST*-mutated cells. While peroxisomes accumulate distally, mitochondria accumulate around the nucleus of olfactory neural stem cells^13^. Similarly, mitochondria accumulate around the neuronal cell body in the brain and spinal cords of humans with *SPAST* mutations^32^ also suggestive of a mitochondrial transport deficit. Mitochondria transport was impaired in neurons generated from induced pluripotent cells of patients with *SPAST* mutations, in which the frequency and speed of motile mitochondria were reduced, with a reduced number of retrogradely travelling mitochondria^11^,^12^. Given the involvement of mitochondria in the regulation of redox state at many levels^33^, it is plausible that impairment of mitochondria transport could also contribute to the oxidative stress of the patient-derived cells of the present study.

Through these observations we have developed a working hypothesis for the genetic and cellular mechanisms leading to cell death in *SPAST*-mutated patient cells. The patients in this study had several different *SPAST* mutations but similar haploinsuffiency in both isoforms of spastin^13^. Individual patient cells behaved as a group in all assays, here and previously, independent of any specific genetic mutation^13^,^15^. This supports *SPAST* haploinsuffiency as the causative genetic mechanism. This leads to spastin insufficiency and the reduction in acetylated α-tubulin^13^. The lower availability of stable microtubules reduces the number of interactions between peroxisomes and microtubules and so reduces the number of motile peroxisomes and hence reduces the average speed of the peroxisome population. We hypothesise that the resultant oxidative stress is caused by the deficit in peroxisome turnover resulting from this transport deficit. This is supported indirectly by the observation that epothilone D restores hydrogen peroxide-induced oxidative stress at the same doses that restore acetylated α-tubulin and peroxisome transport in patient cells. Mitochondrial function may also be compromised by deficits in microtubule-dependent transport and could further contribute to oxidative stress in patient cells.

## Materials and Methods

### Participants

HSP patients involved in this study were examined by a neurologist, experienced in movement disorders (CMS). All patients involved in this study exhibited typical *SPAST* HSP clinical symptoms. Details of their clinical phenotypes are mentioned elsewhere^2^. Details of mutations and related patient information and also information related to healthy controls are listed in Table 1. Olfactory mucosa biopsies were obtained with the informed and written consent of the subjects. The biopsies were performed as described^34^,^35^. All procedures were carried out in accordance with the human ethics committee of Griffith University and the Northern Sydney and Central Coast Human Research Ethics Committee, according to guidelines of the National Health and Medical Research Council of Australia.

### Olfactory neurosphere-derived stem cell culture

The cell lines used in this study are the same as those used previously (Abrahamsen *et al.*^13^; Fan *et al.*^15^). Olfactory neurosphere-derived stem cells (ONS cells) were derived from nasal biopsies of patients and healthy controls using our previously published techniques^18^,^35^. All cultures were grown in ONS cell culture media i.e. Dulbecco’s Modified Eagle Medium (DMEM)/F12 (Gibco) with 10% fetal bovine serum at 37°C and 5% CO^2^ before being plated for assays.

### Neuronal induction and Cytochalasin D treatment

To differentiate immature neurons with axon-like processes, ONS cells were seeded in 96-well glass bottom plates (Matriplate; MGB096-1-2-LG-L; Matrical, Inc, 2000 cells/well) pre-treated with poly-L-lysine (10 mg/ml; Sigma) cultured in ONS cell culture conditions. After cell attachment for 24 hours, neural differentiation was initiated by replacing the ONS cell culture media with neural induction media. For 5 days, the cells were cultured in neural induction medium (Neurobasal medium with 1% B-27 and 1% Glutamax; Life Technologies) with medium change every other day. From about day 6, when cells exhibit long processes, they were treated for 4 days with medium supplemented with Cytochalasin D (1 μg/ml; Life Technologies), which depolymerises filamentous actin, with medium change every second day. No difference was observed in the differentiation efficiency of patient and control-derived cells.

### Immunostaining of cytoskeletal markers

Immunocytochemistry was used to verify the cytoskeletal proteins (microtubules and actin) in undifferentiated and differentiated ONS cells. For microtubule immunostaining, undifferentiated and differentiated cells were fixed in 4% paraformaldehyde for 10 minutes at room temperature, permeabilized with 0.1% Triton X-100 in HBSS containing 3% bovine serum albumin (Sigma) for 30 minutes, and incubated with an antibody against acetylated α-tubulin (1:1000; ab24610, Abcam) for 1 hour at room temperature. Cells were then washed twice in phosphate-buffered saline (PBS, pH 7.4, Gibco Life Technologies) and incubated with a secondary antibody (1:400; goat anti-mouse Alexa Fluor 546, A11003; Life Technologies) for 1 hour at room temperature. For actin labelling, cells were washed twice in PBS and stained with phalloidin (1:100; Alexa Fluor 488^®^ phalloidin; Life Technologies) for 20 minutes at room temperature. Negative controls were included in each run, in which primary or secondary antibodies were not included. For nucleus labelling cells were then washed twice and stained with DAPI (1:1000; Life Technologies) for 10 minutes at room temperature.

### Peroxisome imaging and analysis in living cells

Cells were cultured in 96-well glass bottom plates (Matriplate; MGB096-1-2-LG-L; Matrical, Inc; 2000 cells/well) coated with poly-L-lysine (10 mg/ml) and transduced for 12 hours with a live-cell GFP peroxisome probe (1:200; C10604, CellLight Peroxisome-GFP BacMam 2.0; Life Technologies). For peroxisome imaging, live-cell time-lapse imaging of GFP fluorescent peroxisomes was performed with the Zeiss AxioObserver Z1 microscope (Zeiss) using a Zeiss AxioCamHs camera (Zeiss) under high magnification (oil immersion objective, 630× total magnification). The microscope had a chamber to maintain optimal temperature (37 °C) and CO_2_ (5%) levels throughout the imaging process. The Zeiss AxioVision software (AxioVs40 V 4.8.2.0(Zeiss)) was used to capture time-lapse movies with three dimensional z-stack images captured every 2 seconds, for a total duration of 4 minutes. The exposure period was constant throughout the experiment. For peroxisome movement analysis, the movies were quantified using a semi-automated image analysis software (Imaris; Bitplane) as described previously^13^. Briefly, the three dimensional images captured each 2 seconds were collapsed into a single two dimensional maximum brightness image in which the peroxisomes were identified based on diameter and fluorescence intensity. To avoid observer bias, the image analysis was automated. The peroxisome movement between sequential images was tracked by the image analysis software, which calculated mean speed (μm/s) of each peroxisome tracked. Peroxisomes not tracked for the entire observation period were deleted from the analysis. For peroxisome distribution analysis, the first frame of every time-lapse movie was used. Peroxisomes along 120 μm of axon-like processes starting 20 μm from cell bodies were quantified. For representative images of peroxisome distribution in fixed cells (shown in Fig. 2B,E), peroxisomes were detected by immunofluorescence using a rabbit antibody to mouse peroxisomal membrane protein PEX14 (1:1000 dilution)^36^,^37^.

### Oxidative stress analysis

For baseline measurement of oxidative stress levels, cells were cultured in poly-L-lysine coated 96-well glass bottom plates (Matriplate; MGB096-1-2-LG-L; Matrical, Inc ; 2000 cells/well). To evaluate the effect of H_2_O_2_ stress, the cells were exposed to 50 μM H_2_O_2_ for 1 hour before measuring 4HNE levels. Cells were fixed in 4% paraformaldehyde for 10 minutes at room temperature, permeabilized with 0.1% Triton X-100 in HBSS containing 3% bovine serum albumin (Sigma) for 30 minutes, incubated with an antibody against 4HNE (1:250; HNE11-S, Alpha Diagnostic International) for 2 hours at room temperature. Cells were then washed twice in phosphate-buffered saline (PBS, pH 7.4, Gibco Life Technologies) and incubated with a secondary antibody (1:400; goat anti-rabbit Alexa Fluor 488; Life Technologies) for 1 hour at room temperature. The cell cytoplasm was stained with HCS Cellmask deep red (1:5000 in PBS; H32721, Molecular Probes, Life Technologies) for 30 minutes. The cell nucleus was stained with DAPI (1:1000; Life Technologies) for 10 minutes at room temperature. Cells were imaged with an automated microscope (Operetta High Content Imaging System, Perkin Elmer). Images were captured at 13 locations in each well of the 96 plate (with duplicate plates) with a 20× objective at required wavelengths (488 nm, 4HNE; 647 nm, CellMask; 350 nm, DAPI). To obviate observer bias and experimental variability, the image analysis was automated with the same parameters for every image using Harmony High Content Analysis Software (Perkin Elmer). The software identifies the cells using the nucleus DAPI fluorescence and identifies the cell area using the cytoplasm Cell Mask fluorescence. The fluorescence intensity of 4HNE is quantified in the cell cytoplasm between the nucleus and cell edge. The analysis provides a 4HNE mean fluorescence value based on at least 1000 cells per cell line.

To evaluate the effect of epothilone D (EpoD), ONS cells were seeded in poly-L-lysine pre-coated 96-well plates (2500 cells/well) in duplicate. 24 hours after seeding, the cells were treated with epothilone D (2nM in culture medium; R&S Pharmchem co.ltd, China) for 7 days. This concentration and duration of EpoD treatment was shown previously not to be toxic for the cells^15^. After EpoD treatment, the cells were exposed to 50 μM H_2_O_2_ for 1 hour before quantifying 4HNE levels. After EpoD treatment cells were also assessed for cell viability (MTS assay; CellTiter 96® AQueous One Solution Cell Proliferation Assay, G3580, Promega) and ATP production (ATPlite assay; 6016941, Perkin Elmer). The cells were incubated in 125 μM H_2_O_2_ for 8hours and MTS and ATPlite assays were carried out according to manufacturers’ instructions.

### Statistical analysis

All data are expressed as mean ± SEM. Repeated measures analysis of variance was done using SPSS Statistics Version 22 (IBM). Student’s t-tests and Chi-square analysis was done with Prizm (GraphPad), which was also used for graphing the data including the frequency distribution graphs. Quantile regression analysis was undertaken in Stata IC Version 12.1 software (STATA Corp. Texas, USA). For all statistical analyses the alpha value was 0.05.

## Additional Information

**How to cite this article**: Wali, G. *et al.* Mechanism of impaired microtubule-dependent peroxisome trafficking and oxidative stress in *SPAST*-mutated cells from patients with Hereditary Spastic Paraplegia. *Sci. Rep.* **6**, 27004; doi: 10.1038/srep27004 (2016).

## Figures and Tables

**Table 1:** List of patient and control participants.

Cell line ID	Short ID	Sex	Age at biopsy	Mutation	Exon/Intron
HSP patients with *SPAST* mutations					
610070001	H701	M	64	c.1413 + 3_1413 + 6del	Intron 11
610080001	H801	F	46	p.E464D; c.1392 A > T	Exon 11
610080002	H802	F	50	p.L195V; c.583C > G	Exon 3
610080003	H803	M	51	p.L195V; c.583C > G	Exon 3
610080006	H806	M	57	p.E366K; c.1096G > A	Exon 7
Healthy controls					
100080001	C801	F	55		
100080002	C802	M	59		
100080003	C803	F	66		
100080013	C813	M	64		
100080015	C815	F	64		

Genetic analysis of patients previously reported^2^.
